# Deep Learning in the Classification of Stage of Liver Fibrosis in Chronic Hepatitis B with Magnetic Resonance ADC Images

**DOI:** 10.1155/2021/2015780

**Published:** 2021-12-22

**Authors:** Ziquan Zhu, Daoyan Lv, Xin Zhang, Shui-Hua Wang, Guijuan Zhu

**Affiliations:** ^1^Department of Civil Engineering, University of Florida, Gainesville, FL, USA; ^2^Department of Medical Imaging, Huai'an First Hospital, Huai'an, Jiangsu, China; ^3^Department of Medical Imaging, The Fourth People's Hospital of Huai'an, Huai'an, Jiangsu, China; ^4^School of Architecture Building and Civil Engineering, Loughborough University, Loughborough LE11 3TU, UK

## Abstract

Liver fibrosis in chronic hepatitis B is the pathological repair response of the liver to chronic injury, which is a key step in the development of various chronic liver diseases to cirrhosis and an important link affecting the prognosis of chronic liver diseases. The further development of liver fibrosis in chronic hepatitis B can lead to the disorder of hepatic lobule structure, nodular regeneration of hepatocytes, formation of a pseudolobular structure, namely, cirrhosis, clinical manifestations of liver dysfunction, and portal hypertension. So far, the diagnosis of liver fibrosis in chronic hepatitis B has been made manually by doctors. However, this is very subjective and boring for doctors. Doctors are likely to be interfered with by external factors, such as fatigue and lack of sleep. This paper proposed a 5-layer deep convolution neural network structure for the automatic classification of liver fibrosis in chronic hepatitis B. In the 5-layer deep convolution neural network structure, there were three convolution layers and two fully connected layers, and each convolution layer was connected with a pooling layer. 123 ADC images were collected, and the following results were obtained: the accuracy, sensitivity, specificity, precision, *F*1, MCC, and FMI were 88.13% ± 1.47%, 81.45% ± 3.69%, 91.12% ± 1.72%, 80.49% ± 2.94%, 80.90% ± 2.39%, 72.36% ± 3.39%, and 80.94% ± 2.37%, respectively.

## 1. Introduction

Liver fibrosis in chronic hepatitis B is caused by the excessive accumulation of extracellular matrix proteins, including collagen, that occurs in most types of chronic liver diseases [1]. Liver fibrosis in chronic hepatitis B is the pathological repair response of the liver to chronic injury, which is a key step in the development of various chronic liver diseases to cirrhosis and a vital link affecting the prognosis of chronic liver diseases. The further development of liver fibrosis in chronic hepatitis B can lead to the disorder of hepatic lobule structure, nodular regeneration of hepatocytes, formation of a pseudolobular structure, namely, cirrhosis, clinical manifestations of liver dysfunction, and portal hypertension. Liver fibrosis is histologically reversible, and cirrhosis is difficult to reverse but can be reversed in a few cases.

So far, there are three main methods to diagnose liver fibrosis in chronic hepatitis B. The first is the imaging diagnosis of liver fibrosis in chronic hepatitis B [2]: some signs of liver fibrosis can be found by B-ultrasound, MRI, spiral CT, and color Doppler diagnoses, such as irregular or nodular liver outline, changes of liver parenchymal signal, irregular or nodular shape, an increase of spleen thickness, widening of the portal vein, and spleen vein. However, these influential diagnoses cannot make a clear diagnosis of liver fibrosis and fibrosis degree, so it is often used as the auxiliary diagnosis index in clinic. The second is the pathological diagnosis of liver fibrosis in chronic hepatitis B [3]: clinical-pathological diagnosis of liver fibrosis can not only diagnose liver fibrosis but also understand the degree of development of liver fibrosis and potential liver damage. The third is the diagnosis of liver fibrosis in chronic hepatitis B by serum indicators [4]: serum index is the most widely studied method for the diagnosis of liver fibrosis in chronic hepatitis B. There are hyaluronic acid, III type procollagen, para-type collagen, and laminin in the diagnosis of liver fibrosis in chronic hepatitis B. Through the measurement and judgment of its detection value, it is of great value to the diagnosis of liver fibrosis in chronic hepatitis B and the measurement of the degree of liver fiber in chronic hepatitis B. However, the serological indexes are not completely corresponding to the pathological changes of liver fibrosis in chronic hepatitis B at present, so attention should be paid to the differentiation in diagnosis.

The diagnosis of liver fibrosis in chronic hepatitis B is carried out manually by doctors. However, this is very subjective and boring for doctors. Doctors are likely to be interfered with by external factors, such as fatigue, lack of sleep, and so on. With the continuous development of artificial intelligence and computer vision, computer technology has been applied to various fields, such as the analysis of medical images [5]. Subramaniam et al. [6] used CNN to segment and diagnose medical images. Kim et al. [7] used the denoising CNN (DnCNN) method and trained the network using regular-dose images as ground truth and low-dose images as input. Li et al. [8] proposed an FCN model similar to the u-net structure to regress MR images to CT images. Li et al. [9] proposed a new region-based convolution neural network framework for multitask prediction using an epithelial network header and hierarchical network header. Chen et al. [10] proposed a new CNN architecture, called dense res-induction network (DRINET), to improve the convolution layer to learn the characteristics of medical images. Gu et al. [11] proposed a comprehensive attention-based CNN (CA-Net) for more accurate and explainable medical image segmentation that is aware of the most important spatial positions, channels, and scales at the same time. Xiao et al. [12] proposed a Multiscale Receptive Field Convolution Neural Network (MRF-CNN) for the segmentation of the liver portal areas in hematoxylin and eosin- (H&E-) stained whole slide images (WSIs). Yu et al. [13] proposed a new liver fibrosis detecting algorithm based on the ultrasound echo amplitude analysis and deep learning to classify normal and fibrosis tissue in computer simulation data. Reddy et al. [14] proposed a novel CAD framework using convolution neural networks and transfer learning (pretrained VGG-16 model).

This paper proposed a 5-layer deep convolution neural network structure for the automatic classification of liver fibrosis in chronic hepatitis B. This paper's main innovations and contributions are as follows:

- We proposed an automatic classification method of liver fibrosis in chronic hepatitis B
- We used batch normalization to make the model training more stable and avoid gradient explosion

The remaining structure of this paper is as follows: Section 2 introduces the materials, classification, methods, and CNN structure are given in Section 3, Section 4 mainly discusses the experimental results, and Section 5 is about the conclusion, the shortcomings of this paper, and the future research direction.

## 2. Materials

**Figure 1:**
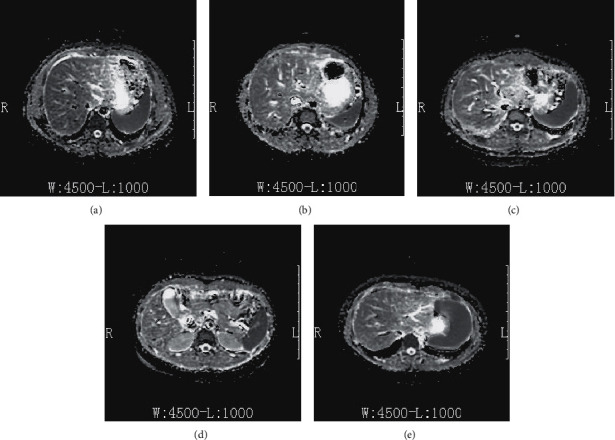
ADC images. (a) *F*0. (b) *F*1. (c) *F*2. (d) *F*3. (e) *F*4.

A total of 123 ADC images were collected from local hospitals with the full knowledge and consent of the patients. All the collected ADC images were divided into stages of *F*0, *F*1, *F*2, *F*3, and *F*4 according to the internationally used METAVIR method, as shown in Figure 1.

The 123 ADC images of patients with chronic hepatitis B were divided into *F*0–*F*4, among which *F*0 had 12 patient ADC images, *F*1 had 26 patient ADC images, *F*2 had 20 patient ADC images, *F*3 had 26 patient ADC images, and *F*4 had 39 patient ADC images, as shown in Table 1. Experienced doctors reconfirmed the identification and classification of all ADC images. This paper used the method binary classification, *F*0 and *F*1 as one positive group and *F*2, *F*3, and *F*4 as one negative group.

## 3. Methodology

**Figure 2:**
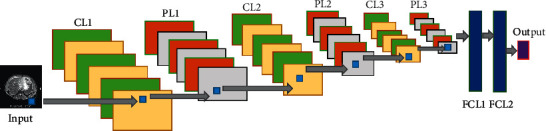
The flowchart of our model.

This paper mainly proposed a 5-layer deep convolution neural network structure for the automatic classification of liver fibrosis in chronic hepatitis B. Since the neural network was proposed, it has been optimized and deepened by researchers [5]. In the 2012 ImageNet Large-Scale Visual Recognition Challenge (ILSVRC), AlexNet [15] won the championship. Two years later, GoogLeNet [16] won the ILSVRC. In 2014, researchers developed a new deep convolution neural network structure: VGG [17]. The proposed 5-layer deep convolution neural network structure is composed of the input layer, three convolution layers, three pooling layers, two fully connected layers, and the output layer, as shown in Figure 2.

### 3.1. Convolution

The convolution layer is one of the essential parts of a deep convolution neural network (DCNN). In the DCNN, the convolution layer implements a 2D convolution for the 3D input and 3D filter, since the channels of both input and filter are the same [18]. The convolution layer has three main characteristics. First, the parameters of the convolution layer are composed of a set of learnable filters. Each filter is small in space (width and height), but the depth is consistent with the input data. Second, it can be seen as an output of a neuron. Neurons only observe a small part of the input data and share parameters with all neurons on the left and right sides of the space. Third, the convolution layer can reduce the number of parameters. Because convolution has the characteristic of “weight sharing,” it can reduce the calculation cost and prevent overfitting due to too many parameters.

In the DCNN, the working principle of the convolution layer is the filter scans the input for convolution operation to extract features [19]. Its specific operation is the filter scans the input from left to right and from top to bottom (find the same part as the filter in the input), then multiplies the input and the filter, and then, sums up to get the output.

**Figure 3:**
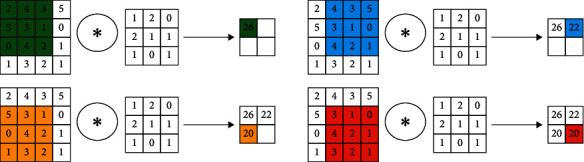
Convolution operation.

As shown in Figure 3, the input matrix size is 4 × 4, the filter matrix size is 3 × 3, and the output matrix size is 2 × 2. We assume there is an input size with *W*_*k*_ × *H*_*k*_ × *D*_*k*_. *W*_*k*_ is the width of the input, *H*_*k*_ is the height of the input, and *D*_*k*_ is the depth of the input. *F*_*w*_ × *F*_*h*_ × *F*_*d*_. *F*_*w*_ is the width of the filter, *F*_*h*_ is the height of the filter, and *F*_*d*_ is the depth of the filter. The number of filters is generally not certain. Researchers usually determine the number of filters by experience. The output is calculated as follows:

(1) $$\begin{array}{c} W_{k+1}=\frac{W_{k}-F_{w}+2B}{Q}+1,\\ H_{k+1}=\frac{H_{k}-F_{h}+2B}{Q}+1,\\ D_{k+1}=M. \end{array}$$

**Figure 4:**
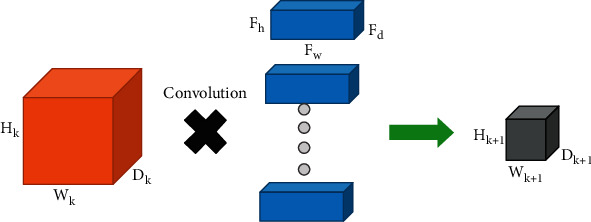
Convolution layer flow chart.

In the abovementioned formula, the size of the output is *W*_*k*+1_ × *H*_*k*+1_ × *D*_*k*+1_, *B* represents the padding, *Q* represents the stride, and *M* denotes the number of filters. The flow chart of the convolution layer is shown in Figure 4.

### 3.2. Pooling

A pooling layer is usually added after one or more convolution layers in the deep convolution neural network. The pooling layer operation does not need a specific kernel. The pooling layer has two advantages: (i) helping to obtain invariance to translation and (ii) reducing the dimension to reduce the amount of calculation [20]. Two pooling layers are commonly used: max pooling and average pooling.

**Figure 5:**
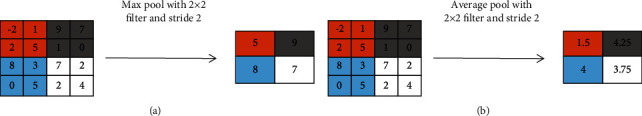
Pooling layer. (a) Max pooling. (b) Average pooling.

Figure 5(a) shows that this is the max pooling that selects the maximum value within the pool region. The width and height of the pooling layer will be reduced by half with a stride of 2. The output size of the pooling operation is a matrix with 2 × 2, while the input size is a matrix with 4 × 4. Pooling operation makes the dimension of input size greatly smaller.

As shown in Figure 5(b), the working principle of average pooling is similar to that of maximum pooling, but the average value replaces the maximum value.

Suppose a rectangular region *R*_*ij*_ is given, where *i* is the number of rows and *j* is the number of columns. The max pooling formula is as follows:

(2) $$\begin{array}{c} m_{ij}=\max x_{pq},\;p=1,\ldots ,i\text{ and }q=1,\ldots ,j, \end{array}$$

where *m*_*ij*_ represents the output value of the rectangular region *R*_*ij*_ by the operation of the max pooling and *x*_*pq*_ represents the element at (*p*, *q*) in the rectangular region *R*_*ij*_.

The average pooling (AP) formula is as follows:

(3) $$\begin{array}{c} a_{ij}=\;\frac{1}{\left|R_{ij}\right|}\sum x_{pq},\;p=1,\ldots ,i\text{ and }q=1,\ldots ,j, \end{array}$$

where *a*_*ij*_ represents the output value of the rectangular region *R*_*ij*_ by the operation of the average pooling, *x*_*pq*_ represents the element at (*p*, *q*) in the rectangular region *R*_*ij*_, and |*R*_*ij*_| represents the number of elements in the rectangular region *R*_*ij*_.

### 3.3. Batch Normalization

For the general neural training model, data standardization has been able to complete the training. However, with the increase of the number of layers and the updating of the parameters in each layer, the results closer to the output layer would change greatly. It is challenging to train deep convolution neural networks effectively [21]. During the model training, batch normalization (BN) uses the mean and standard deviation of small batch to adjust the intermediate output of the neural network continuously [22] so that the value of the intermediate output of the whole neural network in each layer is more stable.

Firstly, the batch *B* is set as

(4) $$\begin{array}{c} B=\;\left\{x_{1},x_{2},\ldots ,x_{n}\right\}, \end{array}$$

where *n* is the number of elements in the batch *B*.

Then, the mean value of the batch *B* is calculated as follows:

(5) $$\begin{array}{c} \mu_{B}\leftarrow \frac{1}{n}\sum_{i=1}^{n}x_{i}. \end{array}$$

The variance is calculated as follows:

(6) $$\begin{array}{c} \sigma_{B}^{2}\leftarrow \frac{1}{n}\sum_{i=1}^{n}{\left(x_{i}-\mu_{B}\right)}^{2}. \end{array}$$

After calculating the mean and variance, the standardized calculation is carried out:

(7) $$\begin{array}{c} z_{i}\leftarrow \frac{x_{i}-\mu_{B}}{\sqrt{\sigma_{B}^{2}+\in \;}}. \end{array}$$

∈(∈>0) is a very small constant, which guarantees that the denominator is greater than 0. Based on the abovementioned standardization, two model parameters (scale parameter *γ* and shift parameter *β*) are introduced into the batch normalization layer to get the output:

(8) $$\begin{array}{c} y_{i}⟵\gamma z_{i}+\beta . \end{array}$$

### 3.4. Rectified Linear Unit

**Figure 6:**
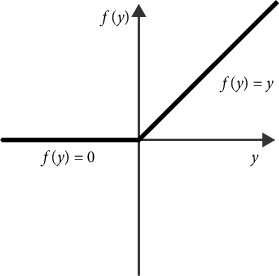
ReLU.

The operation of the activation function is to activate some neurons in the neural network and transmit the activation information to the next layer of the neural network. The neural network can solve nonlinear problems because the activation function adds nonlinear factors, which makes up for the expressive power of the linear model and preserves and maps the “characteristics of activated neurons” to the next layer through functions. In this paper, we used the ReLU function, as shown in Figure 6.

It can be seen from Figure 6 that ReLU is hard saturated when *y* ≤ 0. When *y* > 0, the ReLU can keep the gradient unchanged. The formula is as follows:

(9) $$\begin{array}{c} \text{ReLU}\left(y\right)=\left\{\begin{array}{cc} y, & \text{if }y>0,\\ 0, & \text{ if }y\leq 0. \end{array}\right) \end{array}$$

From Figure 6 and formula (9), we can see that the ReLU activation function has several advantages: (i) in the case of backpropagation, ReLU can avoid the problem of gradient vanishing, (ii) ReLU makes the output of some neurons zero, which leads to the sparsity of the network, reduces the interdependence of parameters, and alleviates the overfitting problem, and (iii) compared with other activation functions, such as tanh and sigmoid, ReLU calculation is very simple.

### 3.5. Structure of DCNN

**Figure 7:**
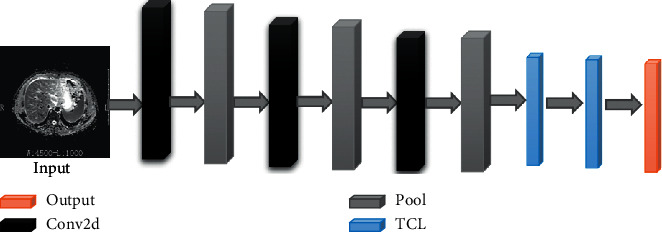
DCNN flow chart.

In this paper, the proposed 5-layer deep convolution neural network structure was composed of three convolution layers and two fully connected layers, as shown in Table 2. Each convolution layer was connected with a pooling layer. Each convolution layer had a different number of convolution kernels. The first convolution layer had 32 convolution kernels, the second had 64 convolution kernels, and the third had 96 convolution kernels. The convolution kernel of each convolution layer was 3 × 3. After three times convolution and pooling calculations, the parameter was 24576. The parameters of the first fully connected layer to the second layer were 24576 × 300. The output of the second fully connected layer was 300 × 2. The flow chart of the DCNN structure is as shown in Figure 7.

### 3.6. Measures

We use 10-fold cross validation to evaluate our model. We set *w* ∈ 1,…, 10, and the confusion matrix is set as

(10) $$\begin{array}{c} M\left(w\right)=\left[\begin{array}{cc} \text{TP}\left(w\right) & \text{FN}\left(w\right)\\ \text{FP}\left(w\right) & \text{TN}\left(w\right) \end{array}\right],\;w\in 1,\ldots ,10, \end{array}$$

where *M*(*w*) is the confusion matrix of the *w*-th run, TP(*w*) represents the true positive of the *w*-th run, FN(*w*) represents the false negative of the *w*-th run, FP(*w*) represents the false positive of the *w*-th run, and TN(*w*) represents the true negative of the *w*-th run.

We can define measures as

(11) $$\begin{array}{c} t_{1}\left(w\right)=\frac{\text{TP}\left(w\right)+\text{TN}\left(w\right)}{\text{TP}\left(w\right)+\text{FP}\left(w\right)+\text{TN}\left(w\right)+\text{FN}\left(w\right)},\;w\in 1,\ldots ,10,\\ t_{2}\left(w\right)=\frac{\text{TP}\left(w\right)}{\text{TP}\left(w\right)+\text{FP}\left(w\right)},\;w\in 1,\ldots ,10,\\ t_{3}\left(w\right)=\frac{\text{TN}\left(w\right)}{\text{FP}\left(w\right)+\text{TN}\left(w\right)},\;w\in 1,\ldots ,10,\\ t_{4}\left(w\right)=\frac{\text{TP}\left(w\right)}{\text{TP}\left(w\right)+\text{FN}\left(w\right)},\;w\in 1,\ldots ,10,\\ t_{5}\left(w\right)=\frac{2\text{TP}\left(w\right)}{2\text{TP}\left(w\right)+\text{FP}\left(w\right)+\text{FN}\left(w\right)},\;w\in 1,\ldots ,10,\\ t_{6}\left(w\right)=\frac{\text{TP}\left(w\right)\times \text{TN}\left(w\right)-\text{FP}\left(w\right)\times \text{FN}\left(w\right)}{\sqrt{\left(\text{TP}\left(w\right)+\text{FP}\left(w\right)\right)\times \left(\text{TP}\left(w\right)+\text{FN}\left(w\right)\right)\times \left(\text{TN}\left(w\right)+\text{FP}\left(w\right)\right)\times \left(\text{TN}\left(w\right)+\text{FN}\left(w\right)\right)}},\;w\in 1,\ldots ,10,\\ t_{7}\left(w\right)=\frac{\text{TP}\left(w\right)}{\sqrt{\left(\text{TP}\left(w\right)+\text{FP}\left(w\right)\right)\times \left(\text{TP}\left(w\right)+\text{FN}\left(w\right)\right)}},\;w\in 1,\ldots ,10, \end{array}$$

where *t*_1_ means accuracy, *t*_2_ means precision, *t*_3_ means specificity, *t*_4_ means sensitivity, *t*_5_ means *F*1, *t*_6_ means MCC, and *t*_7_ means FMI.

We calculate the mean *μ* and standard deviation SD of all the measures (*h* ∈ 1,…, 7):

(12) $$\begin{array}{c} \mu \left(t_{h}\right)=\frac{1}{10}\sum_{w=1}^{10}t_{h}\left(w\right),\\ \text{SD}\left(t_{h}\right)=\sqrt{\frac{1}{10-1}\sum_{w=1}^{10}{\left[t_{h}\left(w\right)-\mu \left(t_{h}\right)\right]}^{2}}. \end{array}$$

**Figure 8:**
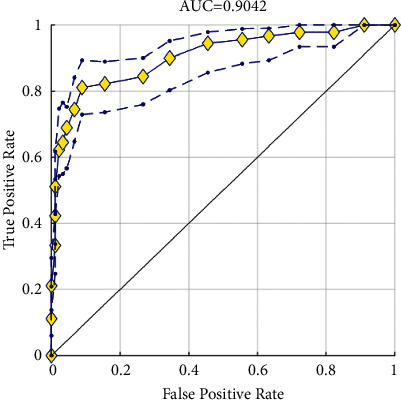
ROC curve.

ROC (receiver operating characteristic) curve: each point on the ROC curve reflects the sensitivity to the same signal stimulation, as shown in Figure 8.

AUC (area under the curve): the area under the ROC curve, between 0.1 and 1. AUC as a numerical value can directly evaluate the quality of the classifier. AUC value is a probability value. The larger the AUC value, the better the current classification algorithm can classify.

### 3.7. Statistics

**Figure 9:**
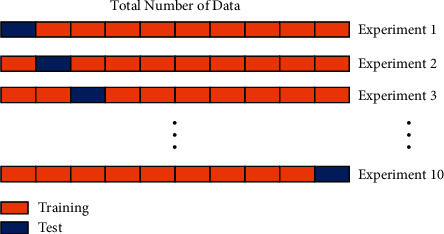
An example of 10-fold cross validation.

10-fold cross validation is used to evaluate the proposed structure, as shown in Figure 9. The dataset is divided into ten parts, nine of which are used as training data and one as test data. Each test gets the corresponding correct rate (or error rate). Ten groups of data are obtained from 10-fold cross validation. The average values of ten groups are used as the evaluation values. To reduce the contingency caused by a single division of training set and test set, the existing dataset is used to partition multiple times. Cross validation is used to reduce contingency and improve generalization ability.

## 4. Experiments

The results of 10-fold cross validation are given in Table 3. Among the ten groups of data, the sensitivity of the ninth group was the highest (86.84), and the sensitivity of the tenth group was the lowest (73.68). The specificity of the tenth group was the highest (95.33), and the specificity of the first group was the lowest (88.24). The highest precision was 83.58 in the tenth group, and the lowest was 75.00 in the first group. The highest accuracy was (90.65) in the ninth group, and the lowest (85.37) accuracy was in the first group. The maximum value of *F*1 was 85.16 in the ninth group, and the minimum value was 92.98 in the first group. The maximum MCC value was 78.37 in the ninth group, and the minimum was 66.27 in the first group. The maximum FMI value was 85.18 in the ninth group, and the minimum was 76.95 in the first group.

It can be concluded from the table that the ninth group of data was the best among the ten groups. The first group of data was the worst, and each data in the first group was the lowest in ten groups.

We introduced the AUC curve and its implications in Section 3.6. Generally speaking, when AUC = 1, it is a perfect classifier; when AUC is [0.85, 0.95], the classifier is very good; when AUC is [0.7, 0.85], the classifier is general; when AUC is [0.5, 0.7], the classifier is bad, when AUC is 0.5, the model has no predictive value; and when AUC <0.5, the classifier is worse than a random guess. As shown in Figure 8, horizontal axis: false positive rate (FPR) and vertical axis: true positive rate (TPR). The AUC value is 0.9042, which proves that our method is highly accurate.

## 5. Conclusions

This paper proposed a 5-layer deep convolution neural network structure for the automatic classification of liver fibrosis in chronic hepatitis B. We used 10-fold cross validation to evaluate the proposed 5-layer deep convolution neural network structure and obtained the following results: the accuracy, sensitivity, specificity, precision, *F*1, MCC, and FMI were 88.13% ± 1.47% 81.45% ± 3.69%, 91.12% ± 1.72%, 80.49% ± 2.94%, 80.90% ± 2.39%, 72.36% ± 3.39%, and 80.94% ± 2.37%, respectively.

The limitations of this study: (i) the training dataset is relatively small. As the number of cases increases and the number of training increases, the system's performance will achieve higher accuracy; (ii) the data collected in this study came from the same hospital. We plan to collect other MRI exams from different centers to evaluate the efficiency of the testing; (iii) we did not compare DCNN structures with the different number of convolution and fully connected layers; and (iv) we did not compare with other approaches.

In the future study, (i) we will collect more data from different sources; (ii) in the next paper, we will do a comparative test on the DCNN structure to get the best DCNN structure.

## Figures and Tables

**Table 1:** Demographics of liver fibrosis in chronic hepatitis B.

	*F*0	*F*1	*F*2	*F*3	*F*4
No. of ADC images	12	26	20	26	39

**Table 2:** DCNN structure.

Layer	Size	Parameters
Input	256 × 256	
Conv. layer 1	64 × 64 × 32	32 3 × 3, pooling size = 2
Conv. layer 2	32 × 32 × 64	64 3 × 3, pooling size = 2
Conv. layer 3	16 × 16 × 96	96 3 × 3, pooling size = 2
FCL1	300 × 1	24576 × 300
FCL2	2 × 1	300 × 2

**Table 3:** Experimental results.

Run	Sen	Spc	Prc	Acc	*F*1	MCC	FMI
1	78.95	88.24	75.00	85.37	76.92	66.27	76.95
2	80.26	92.35	82.43	88.62	81.33	73.16	81.34
3	81.58	91.76	81.58	88.62	81.58	73.34	81.58
4	81.58	88.82	76.54	86.59	78.98	69.22	79.02
5	84.21	89.41	78.05	87.80	81.01	72.16	81.07
6	80.26	91.76	81.33	88.21	80.79	72.29	80.80
7	81.58	91.76	81.58	88.62	81.58	73.34	81.58
8	85.53	91.18	81.25	89.43	83.33	75.66	83.36
9	86.84	92.35	83.54	90.65	85.16	78.37	85.18
10	73.68	95.33	83.58	87.40	78.32	69.76	78.48
MSD	81.45 ± 3.69	91.12 ± 1.72	80.49 ± 2.94	88.13 ± 1.47	80.90 ± 2.39	72.36 ± 3.39	80.94 ± 2.37

## Data Availability

The research data used to support the findings of this study are restricted to protect patient privacy.
